# Genome-Wide Detection for Runs of Homozygosity in Baoshan Pigs Using Whole Genome Resequencing

**DOI:** 10.3390/genes15020233

**Published:** 2024-02-12

**Authors:** Wenjun Li, Xudong Wu, Decai Xiang, Wei Zhang, Lingxiang Wu, Xintong Meng, Jinlong Huo, Zongjun Yin, Guowen Fu, Guiying Zhao

**Affiliations:** 1 College of Animal Science and Technology, Yunnan Agricultural University, Kunming 650201, China; liwenjunmy@163.com (W.L.); wulingxiang11@163.com (L.W.); mxt13384506523@163.com (X.M.); jinlonghuo973@163.com (J.H.); fuguowen1@126.com (G.F.); 2 Institute of Animal Husbandry and Veterinary Medicine, Anhui Academy of Agricultural Sciences, Hefei 230036, China; white_wxd@163.com (X.W.); wzhang1991@126.com (W.Z.); 3 Institute of Pig and Animal Research, Yunnan Academy of Animal Husbandry and Veterinary Science, Kunming 650201, China; askalm@163.com; 4 College of Animal Science and Technology, Anhui Agricultural University, Hefei 230036, China; yinzongjun@ahau.edu.cn

**Keywords:** local breed, whole genome resequencing, runs of homozygosity, inbreeding coefficient, candidate gene

## Abstract

Baoshan pigs (BS) are a local breed in Yunnan Province that may face inbreeding owing to its limited population size. To accurately evaluate the inbreeding level of the BS pig population, we used whole-genome resequencing to identify runs of homozygosity (ROH) regions in BS pigs, calculated the inbreeding coefficient based on pedigree and ROH, and screened candidate genes with important economic traits from ROH islands. A total of 22,633,391 SNPS were obtained from the whole genome of BS pigs, and 201 ROHs were detected from 532,450 SNPS after quality control. The number of medium-length ROH (1–5 Mb) was the highest (98.43%), the number of long ROH (>5 Mb) was the lowest (1.57%), and the inbreeding of BS pigs mainly occurred in distant generations. The inbreeding coefficient *F_ROH_*, calculated based on ROH, was 0.018 ± 0.016, and the *F_PED_*, calculated based on the pedigree, was 0.027 ± 0.028, which were positively correlated. Forty ROH islands were identified, containing 507 genes and 891 QTLs. Several genes were associated with growth and development (*IGFALS*, *PTN, DLX5*, *DKK*1, *WNT2*), meat quality traits (*MC3R*, *ACSM3*, *ECI1*, *CD36*, *ROCK1*, *CACNA2D1*), and reproductive traits (*NPW*, *TSHR*, *BMP7*). This study provides a reference for the protection and utilization of BS pigs.

## 1. Introduction

Pigs are important domestic animals for human societies, as they provide many meat products [1]. In 2023, global pork production was forecast at 114.3 million tons, down 0.2% year-on-year, and global pork consumption was expected to reach 119.845 million tons (United States Department of Agriculture (Washington, DC, USA), USDA, https://www.usda.gov/ (accessed on 5 February 2024)). Hundreds of pig breeds evolved worldwide due to natural selection and artificial selection pressure after the domestication of wild boars, and these pigs meet various needs for humans [2]. However, commercial lean pigs dominate the pig industry with the development of the international market and the globalization of economic activities. Furthermore, the population sizes of indigenous pig breeds are becoming smaller due to the size of commercial grounds. Most indigenous pig farms were built for resource conservation or specific purposes, such as ham production and animal model research [3,4,5].

Yunnan is a multiethnic province in Southwest China rich in animal and plant resources [6]. Baoshan pigs (BS pigs) are bred in Yunnan Province and have a black coat without a saddleback [7]. They exhibit strong adaptability, coarse feeding resistance, good meat quality, and slow growth rate, and have far-reaching effects on local eating habits and ethnic characteristics. Unfortunately, BS pigs have had various unfavorable issues in recent years, and from 2006 to 2016, the number of BS pig sows in the BS pig conservation farm decreased from 976 to 180. In local breeds with small population sizes, one of the biggest problems is the increase in the inbreeding coefficient [8]. Inbreeding depression and the fixation of deleterious mutations in small populations are some of the main genetic factors, combined with non-genetic factors, that are likely to be responsible for the extinction of certain populations [9]. Therefore, to avoid inbreeding depression in BS pigs, a sensitive and accurate estimation of the inbreeding coefficient is necessary.

Runs of homozygosity (ROH) are continuous segments of the genome that arise as a result of inbreeding, resulting in the inheritance of identical haplotypes from parents who share a common ancestor [10]. Artificial or natural selection, population history, genetic drift, effective population size, and breeding models affect the number and distribution of ROH in animals [11,12]. In addition, recombination between homologous chromosomes occurs constantly, and the linkage imbalance between the markers is constantly interrupted, resulting in a decrease in the length of the ROH as the generations increase [13]. ROH is widely used in studies of population history, in examining differences among the population within the same species, in computing inbreeding coefficients, and in analyzing genome-wide associations [8,14,15,16,17,18]. It serves as a valuable tool for understanding genetic diversity and evolutionary processes within and among populations, contributing crucial insights into the broader field of population genetics. The genomic inbreeding coefficient, calculated based on ROH, is closer to the true inbreeding coefficient of an individual, which is more accurate than the expected inbreeding coefficient calculated by traditional genealogy, no longer relies on the accuracy and completeness of the genealogical records, and can accurately reflect the relationship between two gametes. *F_ROH_* is the most effective and accurate method for detecting inbreeding effects, and it is also the closest to the true inbreeding coefficient [15,19,20]. In this study, we aimed to evaluate the genome of BS pigs using whole genome sequencing technology to detect genetic variations within their genomes, in addition to evaluating ROH regions, with the aim of contributing to the development of a BS pig breeding program and providing a reference for the development and use of BS pigs.

## 2. Materials and Methods

This study was approved by the Animal Ethics Committee of Yunnan Agricultural University (approval no. 202103024). Twelve BS pigs were collected from a conservation farm and were not closely related to each other (Shidian County, Baoshan City, Yunnan, China). All DNA samples were sequenced using an Illumina sequencing platform (Illumina, San Diego, CA, USA) at Genedenovo Biotechnology Co., Ltd. (Guangzhou, China) with 10× coverage. In total, 339.12 Gb of raw data from the 12 pig genomes were obtained and submitted to GSA (https://ngdc.cncb.ac.cn/gsa (accessed on 16 September 2021)) under accession number CRA009441. The raw resequencing reads were filtered using the Genome Analysis Toolkit, and quality control standards were set as follows: -Window 4, -filter “QD < 4.0 || FS > 60.0 || MQ < 40.0”, -G_filter “GQ < 20” (QD: Variant Confidence/Quality by Depth; FS: Phred-scaled *p*-value using Fisher’s exact test to detect strand bias; MQ: RMS Mapping Quality; GQ: Genotype Quality). SNP genome coordinates were obtained from the Sus scrofa 11.1 porcine genome reference assembly (https://www.ncbi.nlm.nih.gov (accessed on 20 September 2021)). The analytical procedures for variant calling, SNP filtering, and annotation of filtered SNPs have been described by Li et al. (2009) and Wang et al. (2020) [21,22,23]. 

ROHs were identified for each animal using PLINK v1.07 software using the homozyg command (http://www.cog-genomics.org/plink (20 September 2021)). The following criteria were chosen for ROH estimation in livestock using a medium-density SNP array: (1) the minimum length of the filter input regions was set to 100 kb; (2) one heterozygous and five missing calls were allowed per window to account for genotyping errors; (3) the minimum number of SNPs was set to 50; and (4) the minimum SNP density was set to 1000 bp [24].

The number of ROHs was counted for each 1 Mb length interval(<1 Mb, 1–2 Mb, 2–3 Mb, 3–4 Mb, 4–5 Mb, >5 Mb), and ROH fragments were divided into three categories according to their physical length characteristics: short ROHs (<1 Mb), medium-length ROHs (1–5 Mb), and long ROHs (>5 Mb) [25,26,27]. The proportion of ROHs with different lengths in the experimental pig population and on different chromosomes was counted, and a descriptive analysis was performed.

The inbreeding coefficients *F_ROH_* and *F_PED_* were calculated to evaluate the degree of inbreeding. *F_ROH_* was calculated as the ratio of the total length of the ROH fragment in the genome to the total length of the genome. *F_PED_* uses the path chain method to calculate the inbreeding coefficient of a pedigree. Starting with the earliest genealogically recorded ancestor of the 11BS pigs as the first generation, each BS pig had five generations from the first generation to itself, and the inbreeding coefficient of the first-generation ancestor was treated as zero. SPSS23.0 software was used to conduct a Pearson correlation test for the degree of correlation between the inbreeding coefficients calculated by the two algorithms. *F_ROH_* and *F_PED_* were calculated as follows:

$$F_{ROH}=\sum \frac{L_{ROH}}{L_{AUTO}}$$

where *F_ROH_* is the inbreeding coefficient, *L_ROH_* is the total length of the autosomal ROH interval, and *L_AUTO_* is the total length of the autochromosomes.

$$F_{PED}=\sum \left[{\left(\frac{1}{2}\right)}^{n_{1}+n_{2}+1}\times \left(1+F_{A}\right)\right]$$

*F_PED_* is the inbreeding coefficient of individual X, *n*_1_ represents the algebra of father to common ancestor, *n*_2_ represents the algebra of mother to common ancestor, *F_A_* represents the inbreeding coefficient of common ancestor *A*, and “∑” represents the sum of the values calculated for each path chain.

Taking the population as a unit, the ROH ratio of the SNP sites in the ROH was determined for each SNP site. A Manhattan map was drawn based on the ROH ratio at each SNP site. The ROH ratio where the top 1% was located was taken as the threshold line of the high-frequency SNP, and the ROH island was obtained according to the distribution of SNP sites exceeding the threshold in the genome. The gene contents of the ROH islands were annotated using the annotation database provided by NCBI (https://www.ncbi.nlm.nih.gov (accessed on 23 September 2021)). To further analyze the functions of the identified genes, Gene Ontology (GO)and Kyoto Encyclopedia of Genes and Genomes (KEGG) analyses were performed using the KOBAS 3.0 software (http://kobas.cbi.pku.edu.cn/ (accessed on 23 September 2021)). The items and pathways significantly enriched in GO and KEGG were selected (*p* < 0.05), the genes in the pathways were analyzed using the STRING 12.0 online software for gene interaction, and the interaction results were visualized using Cytoscape_v3.10.1.

The pig Quantitative Trait Locus/Loci (QTL) database (https://www.animalgenome.org/cgi-bin/QTLdb/SS/index (accessed on 24 September 2021)) was used to select all data by bp version (in SS11.1, GFF format), and the ROH island was mapped with the QTL data to obtain QTL character information. Combined with the GO and KEGG enrichment results, candidate genes related to important economic traits in experimental pigs were screened. 

## 3. Results

**Figure 1:**
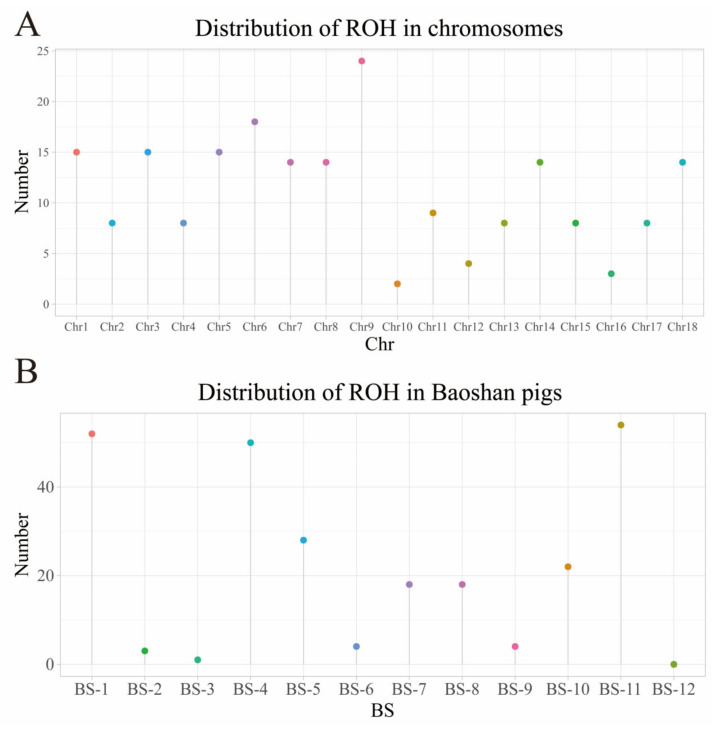
ROH number statistics of Baoshan pigs. (**A**) Distribution of ROH numbers on chromosomes; (**B**) distribution of ROH numbers on individual BS pigs.

A total of 22,633,391 SNPs were detected in the entire BS pig genome, and 532,450 high-quality SNPs were retained for ROH analysis after filtering out low-quality SNPs (Figure S1A). A total of 201 ROH were detected in 12 BS pigs, with an average of 16.75 ROH per sample, distributed on all 18 autosomes, and the number of ROH on Chr9 was the highest (37); Chr10 and Chr16 had the lowest number of ROH (Figure 1A). BS-12 had no ROH fragments, and BS-11 had the highest number of ROH fragments (Figure 1B). 

**Figure 2:**
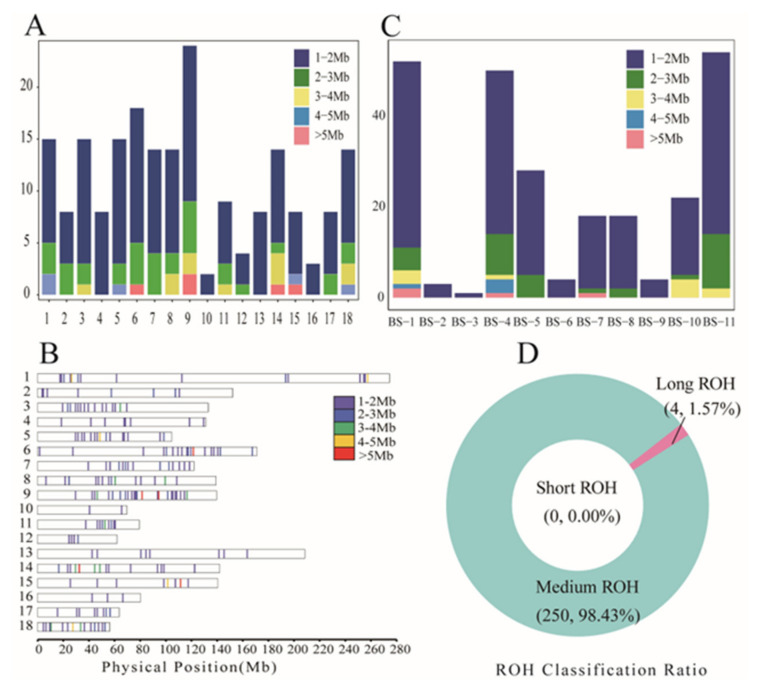
Statistics of ROH length and distribution in BS pigs. (**A**) Distribution of different lengths of ROH on chromosomes; (**B**) position of different lengths of ROH on chromosomes; (**C**) distribution of different lengths of ROH on individual BS pigs; and (**D**) number and proportion of long, medium, and short ROHs.

The length of ROH in BS pigs was categorized into six length intervals of <1 Mb, 1–2 Mb, 2–3 Mb, 3–4 Mb, 4–5 Mb, and >5 Mb, with each length accounting for 0.00%, 79.13%, 13.78%, 3.94%, 1.57%, and 1.57%, respectively (Figure S1B). The overall average length of the ROH was 1.750 ± 0.960 Mb (Figure S1C). ROHs with lengths of 1–2 Mb were distributed across chromosomes, ROHs with lengths >5 Mb were present only on Chr6, Chr9, Chr14, and Chr15 (Figure 2A), and ROHs of all lengths were unevenly distributed across chromosomes (Figure 2B). Similarly, ROHs with lengths of 1–2 Mb were present in all BS pigs, but ROHs with lengths >5 Mb were present only in BS-1, BS-4, and BS-7 pigs (Figure 2C). The highest number of medium-length ROHs (98.43%) and the lowest number of long ROHs (1.57%) were found (Figure 2D), suggesting that inbreeding in BS pigs occurred in distant ancestors.

The average *F_PED_* for the BS pig population was 0.027 ± 0.028, with a maximum value of 0.044 and a minimum value of 0.001. The inbreeding coefficient of each individual was less than 0.100. The average *F_ROH_* for the BS pig population was 0.018 ± 0.016, with a maximum value of 0.094 and a minimum value of 0.004. The inbreeding coefficient of each individual was less than 0.050 (Figure S1D), which indicated that the inbreeding level of BS pigs was low. The correlation coefficient between the two inbreeding coefficients was 0.462, the significance was 0.152, and the correlation was not significant (*p* > 0.050) (Figure S1E).

**Figure 3:**
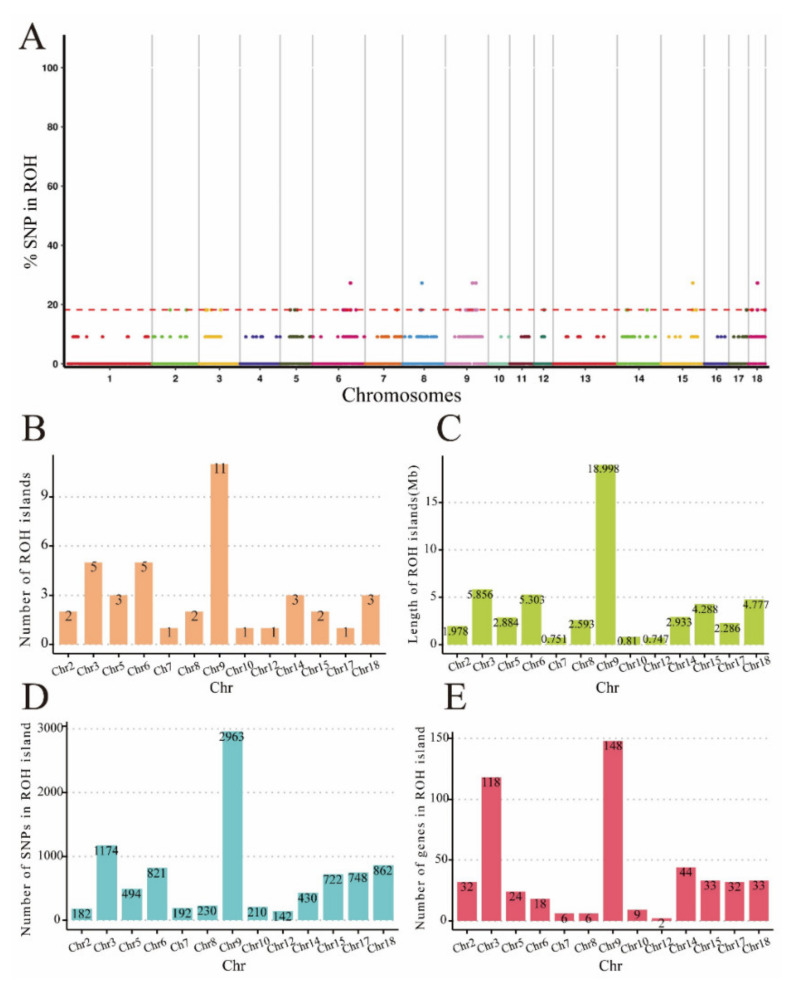
Identification and statistics of ROH islands in BS pigs. (**A**) Identification of ROH islands in BS pigs; the red dashed line in the figure indicates the threshold line and the colored dots overlapping with or above the threshold line indicate the detected ROH islands. (**B**–**E**) Statistics of the distribution of ROH islands on chromosomes in terms of the number of ROH islands in BS pigs, their lengths, the number of SNPs, and the number of genes.

A total of 40 ROH islands containing 507 genes were detected in BS pigs (Tables S1 and S2). ROH islands in BS pigs were distributed on all chromosomes except Chr1, Chr4, Chr11, Chr13, and Chr16. Chromosome 9 possessed the most ROH islands, and the total length of the ROH island on this chromosome was the longest at 18.998 Mb, and contained the highest number of SNPs (Figure 3). 

**Figure 4:**
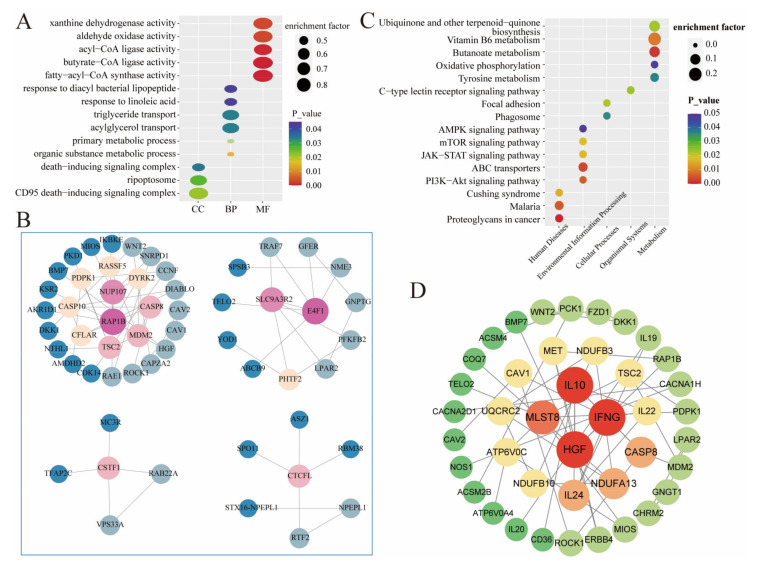
GO and KEGG enrichment and gene interactions in ROH islands. (**A,C**) GO/KEGG enrichment of genes in ROH islands. Horizontal coordinates indicate the first level of classification, vertical coor-dinates indicate the third level of classification, the size of the dots indicates the enrichment factor (the ratio of the number of genes enriched to the island to the total number of all the genes in the entry), the bigger the dots, the more genes enriched, and the color of the dots indicates the signifi-cance, the darker the color, the more significant the relationship. (**B,D**) Network diagram of gene interactions in the GO entry/KEGG pathway. A dot indicates a gene, the size and color of the dot indicates interactions, the larger the dot and the darker the color, the more interactions of the gene, the gene at the center is the one with the most interactions.

Genes were significantly enriched in 219 tertiary entries across all GO entries, including 3 cellular components, 60 biological processes, and 156 molecular functions (Table S3). The genes were mainly related to biological processes, such as organic matter metabolism, lipid substance metabolism, compound metabolism, and molecular functions, such as multiple enzyme activities (ligases, synthetases, and transporter enzymes), substance transport, and substance binding. Some of the significantly enriched entries are shown in Figure 4A. The genes in the significant entries formed a total of four interaction networks, of which 30 genes had interactions centered on *NUP107* and *RAP1B*, 13 genes had interactions centered on *SLC9A3R2* and *E4F1*, 5 genes had interactions centered on the *CSTF1* gene, and 7 genes had interactions centered on the *CTCFL* gene (Figure 4B). The genes were significantly enriched in 16 secondary pathways across all KEGG pathways (Table S4). These included three pathways for Human Diseases, five pathways for Environmental Information Processing, two pathways for Cellular Processes, one pathway for Organismal Systems, and five pathways for metabolism (Figure 4C). The 41 genes in these pathways formed an interaction network centered on 4 genes: *IL10*, *IFNG*, *HGF*, and *MLST8* (Figure 4D).

A total of 891 QTLs were identified by QTL localization in the ROH islands of BS pigs. The search for QTL-related traits revealed that the QTL loci in BS pigs could be classified into four major categories: reproductive performance, growth performance, slaughter and meat quality performance, and health, with 62, 90, 650, and 89 QTL localized in each category, respectively. Most QTLs were localized in slaughter and meat quality performance, accounting for 72.95% of all loci, and were mainly concentrated in the loci of drip loss, muscle and fat content, pH change, and meat color score (Table S5).

## 4. Discussion

Livestock that have been in closed breeding and artificial high-intensity selection environments for a long time have a limited number of good breeding animals, which are used more frequently in actual production. Therefore, inbreeding inevitably occurs, resulting in a reduction in population diversity [28]. The number of purebred BS pigs was maintained only through breeding preservation farms, and the limited population size and frequent use of a small number of boars may have led to inbreeding in BS pigs. Evaluating the level of inbreeding can provide a basis for the future development of breeding programs that are conducive to the conservation and exploitation of BS pigs as a breeding resource.

The ROH is commonly used to assess the proximity of kinship between individuals of livestock, and thus predict the period when inbreeding occurs [29]. The longer the ROH, the closer the kinship between individuals, suggesting that inbreeding occurred in the near-generation ancestor, and vice versa. The shorter the ROH, the further the kinship between individuals, suggesting that inbreeding occurred in the distant-generation ancestor [30,31,32]. The presence of a higher number of long ROHs in the domesticated Asian wild boar population suggests that recent inbreeding has occurred in this population, which may be correlated with the recent population decline in Asian wild boars [33]. Domestic pigs in the Iberian Peninsula have a longer ROH than wild boars, suggesting that inbreeding occurred later in wild boars than in domestic pigs [34]. Wild boars that have experienced population size bottlenecks over the last century have undergone inbreeding, confirming the reliability of this result [35]. Diannan Small-Ear Pigs (DSE) from Yingjiang, Jinping, and Sipsongpanna contained 1122, 1244, and 720 ROHs, respectively. The lengths of ROHs in DSE pigs from the three regions primarily fell within 1–5 Mb, and the number of ROHs within 1–5 Mb accounted for more than 88% of the total number of ROHs in each subgroup, and the percentage of longer ROHs (>10 Mb) did not exceed 3% [36]. Landrace, Songliao, and Yorkshire pigs showed six length types: <5 Mb, 5–10 Mb, 10–20 Mb, 20–40 Mb, and >40 Mb. Although all three pig breeds had the highest number of <5 Mb ROH, the number of long ROH > 5 Mb still accounted for the larger number of long ROH; moreover, the average number of individual ROH in the three pig breeds was 32.99, and the average ROH length was 6.40 Mb [37]. In contrast, the numbers and lengths of ROHs in the BS pigs in this study were smaller than those in DSE, Changbai, Songliao, and Yorkshire pigs, and the BS pigs contained more medium-length ROHs (1–5 Mb) and almost no long ROHs, suggesting that inbreeding in BS pigs occurred in more distant generations. Since Western pig breeds have been selected for many years with high intensity, the number of long ROHs should be much greater than the number of long ROHs in Chinese local pig breeds, but the results were the opposite. Studies have shown that many Chinese pig breeds possess a greater number of ROHs, especially longer ROHs, than Western pig breeds, because the effective population size of Chinese pig breeds was too small [38]. In fact, inbreeding was more common in China owing to the low awareness of pig breed resource conservation in the early days, and small population size was a common problem for most Chinese pig breeds. Despite the declining number of BS pigs in recent years, almost no inbreeding has occurred, suggesting that the conservation of BS pigs has been more effective.

The inbreeding coefficient of pig breeds is an important index to measure the genetic diversity of pig breed populations; the higher the inbreeding coefficient, the more prone it is to inbreeding decline, such as growth retardation, reduced fertility, and weakened disease resistance [39,40]. *F_PED_* and *F_ROH_* are commonly used to calculate the inbreeding coefficients of livestock and poultry; however, inbreeding coefficients calculated using *F_ROH_* are more accurate [41]. The Nero Lucano Pig had an inbreeding coefficient *F_PED_* of 0.057 calculated by genealogy and an inbreeding coefficient *F_ROH_* of 0.39, calculated using ROH. Although *F_PED_* was lower than *F_ROH_*, it was not as accurate as *F_ROH_* because of the incomplete genealogy and the default inbreeding coefficient of 0 for the starting generation, which underestimated the true inbreeding level [42]. The *F_ROH_* of the six native pig breeds from Croatia, Serbia, and Slovenia were closer to the true level of inbreeding than the *F_PED_*, with the highest inbreeding coefficients among the six breeds for the Turopolje pig, with *F_ROH_* and *F_PED_* values of 0.508 and 0.038, respectively [43]. In fact, the absence of genealogical records resulted in inbreeding phenomena that originally occurred in distant generations not being considered, and the originally longer chain of genealogical pathways was artificially shortened; thus, the accuracy of inbreeding coefficient estimates was not as accurate as inbreeding coefficients calculated based on the ROH of genes [44]. *F_PED_* (0.0268) was slightly higher than *F_ROH_* (0.0178) for the BS pig population in this study. Unlike the underestimation of inbreeding levels described above, *F_PED_* overestimated the true inbreeding level. This is because the *F_PED_* was calculated for BS pigs from the fifth generation of ancestors and for the above-mentioned breeds from the third generation of ancestors, and the inbreeding coefficients of ancestors without genealogical records were treated as zero. However, the length of pedigree path chains was different; therefore, the inbreeding coefficients may have been overestimated or underestimated. However, the inbreeding coefficients calculated using both methods were low for BS pigs, indicating low levels of inbreeding. Studies have shown that there is a high positive correlation between *F_PED_* and *F_ROH_* with a correlation coefficient of up to 0.75 for inbreeding occurring within five generations, and a moderately low correlation between *F_PED_* and *F_ROH_* with a correlation coefficient generally lower than 0.5 for inbreeding occurring outside five generations [45]. For example, the correlation coefficients between *F_PED_* and *F_ROH_* in published studies were 0.18–0.37 for Large White pigs, 0.161 for Pietrain pigs, 0.514–0.523 for Duroc pigs, and 0.49–0.54 for Blackbottom pigs, indicating that the two calculations were positively correlated but the correlation was not high [41,46,47,48]. In this study, the correlation coefficient between *F_PED_* and *F_ROH_* in BS pigs was 0.462, which was positively correlated, consistent with the results from the above studies. The correlation was lower than 0.5, indicating that inbreeding in BS pigs occurred prior to the fifth generation of ancestors, which is consistent with the prediction of a previous study that inbreeding in BS pigs occurs in distant generations.

The distribution of ROHs in the genome is population specific, and the identification of ROH islands is considered an effective method for identifying genomic regions under natural or artificial selection [49,50]. QTL localization can reveal the relationship between genes in the ROH island and important traits and provide a basis for the genetic improvement and breeding of pigs. BS pigs are characterized by high fat content, tender meat, unique pork flavor, good reproductive performance, and a slow growth rate. In this study, we screened candidate genes and QTL related to the ROH islands of BS pigs by studying the functions and QTL localization of genes in the characteristics of BS pigs. We screened five genes related to growth performance in BS pigs: *IGFALS*, *PTN*, *DLX5*, *DKK1,* and *WNT2*. Among them, the *IGFALS* gene was mainly enriched in several regulatory pathways, such as growth hormone synthesis, growth factors, and their complex synthesis. It is commonly expressed in pigs and stimulates the growth potential of pigs [51,52,53]. The *PTN* gene is enriched in several processes, such as growth factor activity, cell proliferation and differentiation, and the positive regulation of cell division, and is a candidate gene affecting backfat thickness in several pig breeds.[54].The average daily gain and body weight QTL located on Chr4, Chr7, and Chr9 in BS pigs may be subject to the regulatory effects of these two genes. *DLX5*, *DKK1* and *WNT2* are mainly enriched in the WNT signaling pathway and are involved in bone production, renewal and healing by regulating the expression of osteogenic coregulators, which are capable of influencing body size and thoracic vertebrae number in pigs.[55,56,57,58,59]. BS pigs are classified as large, medium, or small, probably due to differences in the expression and regulatory effects of osteogenic genes that differentiate their body sizes. Second, we screened six genes related to meat mass traits in BS pigs: *MC3R*, *ACSM3*, *ECI1*, *CD36*, *ROCK1*, and *CACNA2D1*. The GO and KEGG annotation results for these genes indicated that they were mainly related to lipid metabolism, lipid synthesis and transport, and fat and muscle differentiation. They promote fat deposition, regulate the muscle-to-fat ratio, affect pork fat content and marbling, etc., and are often used as key genes in the study of meat quality traits. There are more obesity-related QTLs in BS pigs, which are widely clustered on Chr4, Chr6, Chr7 and Chr9, and may be associated with these genes [60,61,62,63,64,65]. The *CACNA2D1* gene was found to overlap with QTL for meat quality traits on porcine Chr9 in an earlier study, and its association with stress syndromes may contribute to the formation of poor-quality pork [66]. The BS porcine QTL for meat quality traits located on Chr9 may be associated with this gene, and is likely to be associated with pale, soft, and exudative meat on the Chr4 QTL. Finally, we screened three genes associated with the reproductive performance of BS pigs: *NPW*, *TSHR*, and *BMP7*. The *NPW* gene was annotated to the neuroactive ligand-receptor interaction and neuropeptide signaling pathways. It is involved in the regulation of reproduction through the promotion of porcine testicular mesenchymal stromal cell production and secretion of testosterone [67]. This may be related to the testicular weight and testosterone level QTL, which are located on Chr5 and Chr7, respectively. The *TSHR* gene regulate seasonal reproductive activities in animals through hypothalamic-pituitary-gonadal axis [68,69]. The *BMP7* gene is mainly enriched in several reproduction-related pathways and entries, such as embryonic placental development, urogenital development, and uterine embryo development. Previous studies have focused on it as a candidate gene related to reproductive traits, such as ovarian function, litter size, number of live-born piglets, and litter weight of piglets [70,71,72]. The QTL related to reproductive performance in BS pigs in this study were mainly concentrated on Chr1, Chr3, and Chr7, especially those related to ovary weight, litter size, and number of stillbirths, which are likely related to the regulatory pathway of *MBP7*. 

In addition, among the 14 genes mentioned above, seven genes, namely, *WTN2*, *DKK1*, *MC3R*, *CD36*, *ROCK1*, *CACNA2D1*, and *BMP7*, interacted with each other, forming an interaction network mainly related to lipid transportation, fat deposition, and meat quality traits. It is likely that the high fat content, tenderness, and adaptability of BS pigs are the result of synergistic effects of these genes. The functions of the above genes were hypothesized by us with reference to gene annotation information and previous studies, which indicated that these genes may influence the production performance of BS pigs. However, further research is required to determine whether they can be used as candidate genes. On the other hand, the sample size of this study is small, which may not be representative and only provides a part of the reference, and we will expand the sample size for research validation in next work.

## 5. Conclusions

In this study, we characterized the occurrence and distribution of ROHs in the genome of BS pigs and screened 14 candidate genes from 40 ROH islands, which were associated with the growth and development, meat quality traits, and reproductive performance of BS pigs; however, the specific biological functions of these genes require further investigation. In this study, we only explored the whole genome ROHs of 12 BS pigs with a relatively small number of samples, which may lack representativeness, and we will follow up by further expanding the number of samples to be sequenced in order to complement and improve our study. 

## Data Availability

The datasets presented in this study can be found in online repositories. The names of the repository/repositories and accession numbers can be found at https://ngdc.cncb.ac.cn/gsa, CRA009441 (accessed on 22 December 2023).

## Supplementary Materials

The following supporting information can be downloaded at https://www.mdpi.com/article/10.3390/genes15020233/s1, Figure S1: Additional information on ROH statistics; Table S1: ROH Information for BS Pigs; Table S2: Genetic information in BS pig islands; Table S3: Information on GO enrichment of genes in the ROH island of BS pigs; Table S4: Information on KEGG enrichment of ROH island genes in BS pigs; Table S5: Information on localization of QTLs in BS pig ROH islands.
