# Supplementary figures and images for: Genome-Wide Detection for Runs of Homozygosity in Baoshan Pigs Using Whole Genome Resequencing
Source: Genes (Basel). 2024 Feb 12;15(2):233. doi: 10.3390/genes15020233 (PMC10887577; doi:10.3390/genes15020233)

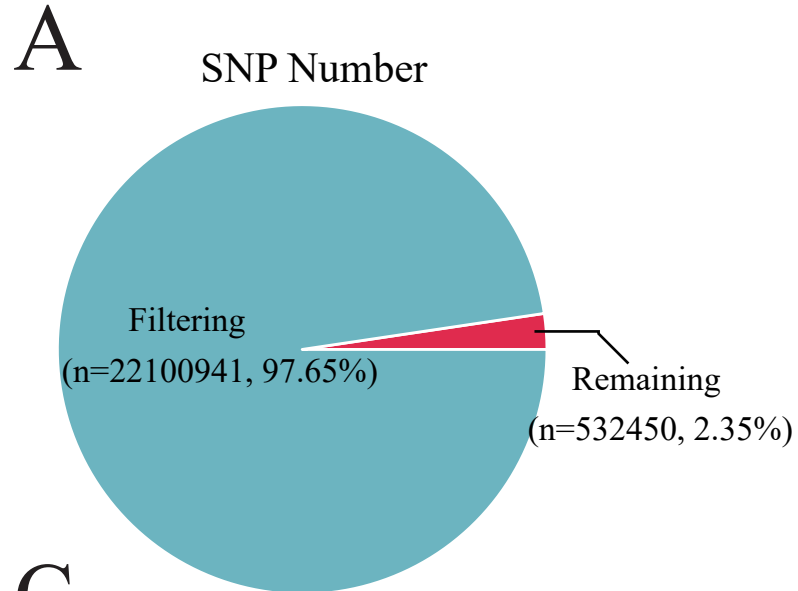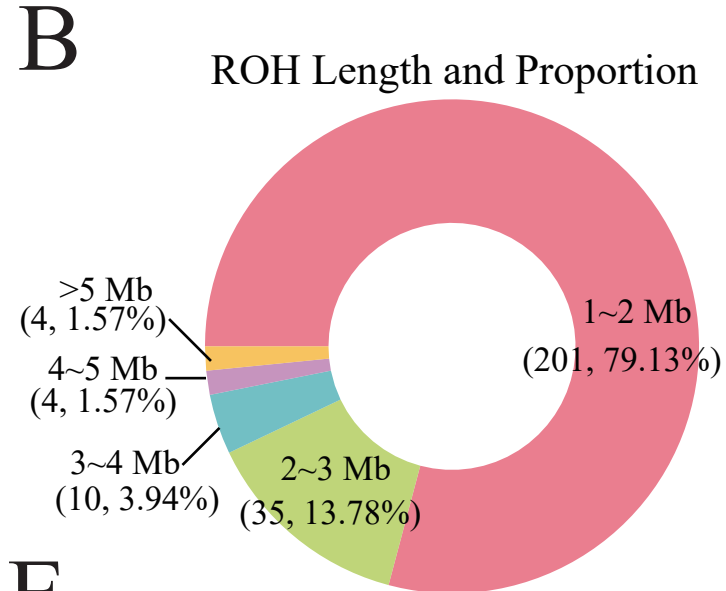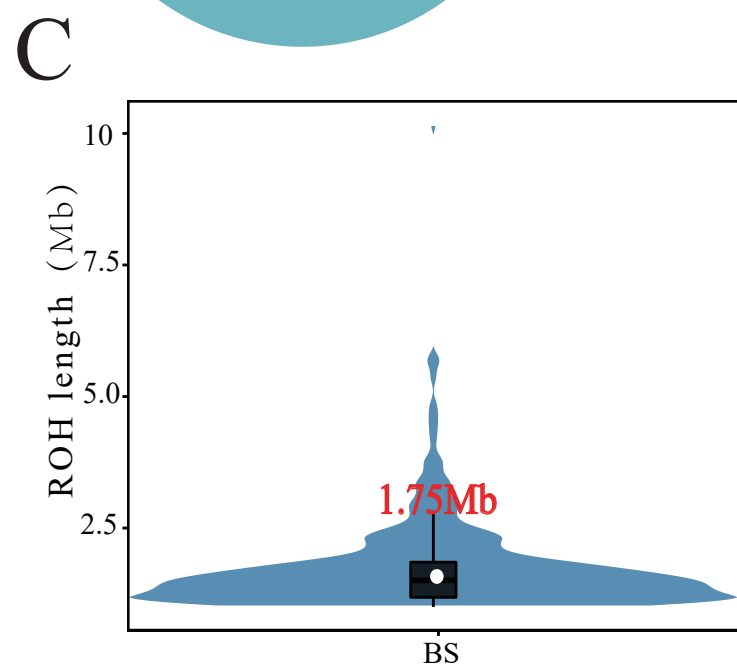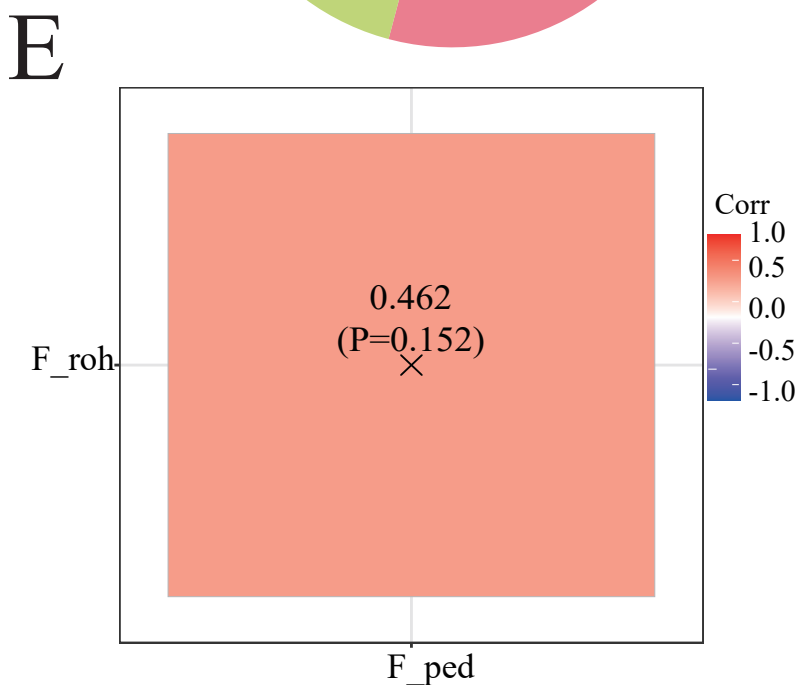

**D** Inbreeding coefficient for BS pigs

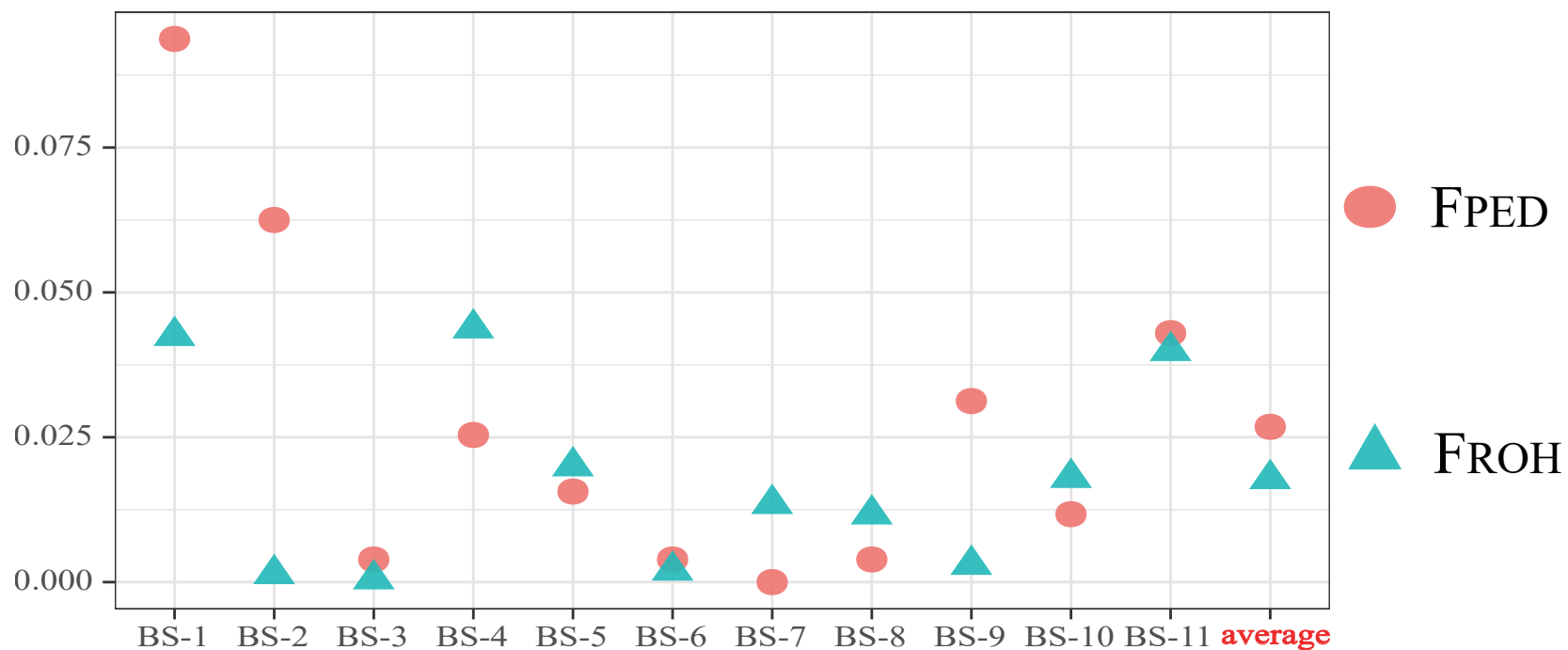

Supplement: Supplementary file 1 [file genes-15-00233-s001.zip › Figure S1 Additional information on ROH statistics.pdf]
